# β-Ketoacyl-acyl Carrier Protein Synthase I (KASI) Plays Crucial Roles in the Plant Growth and Fatty Acids Synthesis in Tobacco

**DOI:** 10.3390/ijms17081287

**Published:** 2016-08-08

**Authors:** Tianquan Yang, Ronghua Xu, Jianghua Chen, Aizhong Liu

**Affiliations:** 1 Key Laboratory of Tropical Plant Resource and Sustainable Use, Xishuangbanna Tropical Botanical Garden, Chinese Academy of Sciences, Xuefu Road 88, Kunming 650223, China; yangtianquan@xtbg.org.cn (T.Y.); jhchen2016@163.com (J.C.); 2 University of Chinese Academy of Science, Beijing 100049, China; 3 College of Life Sciences, Anhui Science and Technology University, Fengyang 233100, China; ronghua.xu08@gmail.com; 4 Key Laboratory of Economic Plants and Biotechnology, Kunming Institute of Botany, Chinese Academy of Sciences, Lanhei Road 132, Kunming 650201, China

**Keywords:** tobacco, 3-ketoacyl-ACP synthase I, over-expression, gene silence, fatty acid synthesis

## Abstract

Fatty acids serve many functions in plants, but the effects of some key genes involved in fatty acids biosynthesis on plants growth and development are not well understood yet. To understand the functions of 3-ketoacyl-acyl-carrier protein synthase I (KASI) in tobacco, we isolated two KASI homologs, which we have designated NtKASI-1 and NtKASI-2. Expression analysis showed that these two *KASI* genes were transcribed constitutively in all tissues examined. Over-expression of *NtKASI-1* in tobacco changed the fatty acid content in leaves, whereas over-expressed lines of *NtKASI-2* exhibited distinct phenotypic features such as slightly variegated leaves and reduction of the fatty acid content in leaves, similar to the silencing plants of *NtKASI-1* gene. Interestingly, the silencing of *NtKASI-2* gene had no discernibly altered phenotypes compared to wild type. The double silencing plants of these two genes enhanced the phenotypic changes during vegetative and reproductive growth compared to wild type. These results uncovered that these two *KASI* genes had the partially functional redundancy, and that the *KASI* genes played a key role in regulating fatty acids synthesis and in mediating plant growth and development in tobacco.

## 1. Introduction

Fatty acids (FAs) are major components for cell or organelle membrane lipids, and precursors of other significant complex molecules including waxes and cutin. Some FAs are also converted into messenger compounds such as jasmonic acid and phosphatidylinositol that play major roles in certain signal transduction pathways [1,2,3]. Furthermore, FAs are used as substrates for the synthesis of storage lipids (triacylglycerols, TAG), particularly in the cotyledon or endosperm of oilseeds, which are important materials for seed germination and food or energy supply for humankind [4,5]. Thus, understanding the biosynthesis of FAs in plants has significance with regard to careful control of plant development as well as practical implications.

During FAs biosynthesis, the first committed step is catalyzed by acetyl-CoA carboxylase (ACCase), which converts acetyl-CoA to malonyl-CoA, and then is condensed by a set of β-ketoacyl-ACP synthases (KASs), resulting in FA chain elongation [6,7,8]. These KASs are crucial for carbon chain condensation and elongation from C4 to C18. In plants, several plastid or chloroplast specific types of KAS, including KASI, KASII, KASIII and KASIV have been characterized in diverse species [9,10,11,12,13,14]. KASIII is responsible for condensing the initial reaction of malonyl-acyl carrier protein and acetyl-CoA, resulting in a C4 FA molecule [15,16]. KASI has high activity when butyryl- to myristyl-ACP (C4:0–C14:0 ACP) is used as the substrate to produce hexanoyl- to palmitoyl-ACP (C6:0–C16:0 ACP), whereas KASII is a key enzyme that catalysis the last condensation reaction of palmitoyl-ACPs to stearoyl-ACPs [17]. KASIV is thought to participate in condensing the medium-chain FA (C10:0 or C12:0 ACP) in certain species such as *Cuphea* [14,18]. In addition, the mitochondrion-specific mtKAS participating in FA synthesis for forming mitochondrial membranes has been isolated and characterized in *Arabidopsis* [19,20], but still very little is known in other plants.

Among these identified KASs, KASI, KASII and KASIII seem to be essential and exist broadly in plants. The genes encoding KASIII and KASII were extensively identified from various plants such as *Spinach oleracea* [21], *Arabidopsis thaliana* [22], *Cuphea wrightii* [23], *Allium porrum* [24], *Pisum sativum* [25], *Helianthus annuus* [12], *Brassica napus* [13] and *Jatropha curcas* [11,26]. In addition, their functions were partially documented in *Jatropha curcas*, sunflower and rapeseeds [11,12,13,26]. In particular, studies have demonstrated that KASIII was a rate-limiting enzyme in TAG accumulation [15] and KASII could cause significant changes of the FA composition in the conversion of from C16 to C18 in TAG biosynthesis [26]. *KASI* genes have been isolated from barley [27], groundnut [9] and rice [28] to date, but the functions of KASI in controlling FAs biosynthesis and regulating plant growth and development remained unclear in plants. Until recently, Wu and Xue demonstrated the functional characterization of *KASI* gene in *Arabidopsis*, and uncovered that *AtKASI* was not only crucial in controlling TAG biosynthesis in both leaf tissues and seeds but also critical in mediating chloroplast formation and division [10]. Besides, the mutants of *OsKASI* reduced fertility and altered the FA composition and contents in roots and seeds, suggesting that *OsKASI* is involved in regulating the root development in rice [28]. Apart from studies in *Arabidopsis* and rice the functions of KASI in controlling FAs biosynthesis and regulating growth and development have not been characterized in other plants.

Tobacco (*Nicotiana tabacum*), as an alternative biofuel plant in recent years, has received a great attention because it possesses potent oil biosynthesis machinery and can accumulate up to 40% oil content in seed. In particular, tobacco leaves have been metabolically engineered as oil-bearing tissues, representing an attractive and promising “energy plant” platform and serving as a plausible system for manufacturing biodiesel production [29,30]. Tobacco oils have been successfully tested for its potential as a fuel for diesel engines [31]. Identification and dissection of key genes encoding the rate-limiting enzymes in FAs and TAG biosynthesis is essential to serve the genetic and metabolic engineering of tobacco for manufacturing oil production. In this study, two tobacco genes encoding KASI were isolated, and their function in mediating FAs and TAG biosynthesis as well as plant growth were characterized. Results obtained in this study provide fundamental and important information for understanding the molecular functions of KASI genes in tobacco.

## 2. Results

### 2.1. Identification of 3-Ketoacyl-ACP Synthase I Gene in Tobacco

After the assembly of EST fragments, two putative KASI fragments were identified with full-length coding regions, named as *NtKASI-1* and *NtKASI-2*, respectively. Subsequently, two full-length cDNA sequences of *NtKASI-1* and *NtKASI-2* were confirmed by the RT-PCR sequencing and were submitted to GenBank (KX033513 and KX033514). NtKASI-1 and NtKASI-2 contain a complete ORF (open reading frame) with 1410 bp and 1404 bp, encoding 469 and 467 amino acids, respectively. These two genes shared high similarities on nucleotide sequence (85%) and amino acid (88%) levels. Besides, there was approximately 83% amino acid sequence identity between tobacco NtKASI-1 and *Arabidopsis* KASI (AtKASI), and 81% sequence identity between tobacco NtKASI-2 and AtKASI. These results showed that tobacco KASIs may have similar functional roles to AtKASI.

**Figure 1:**
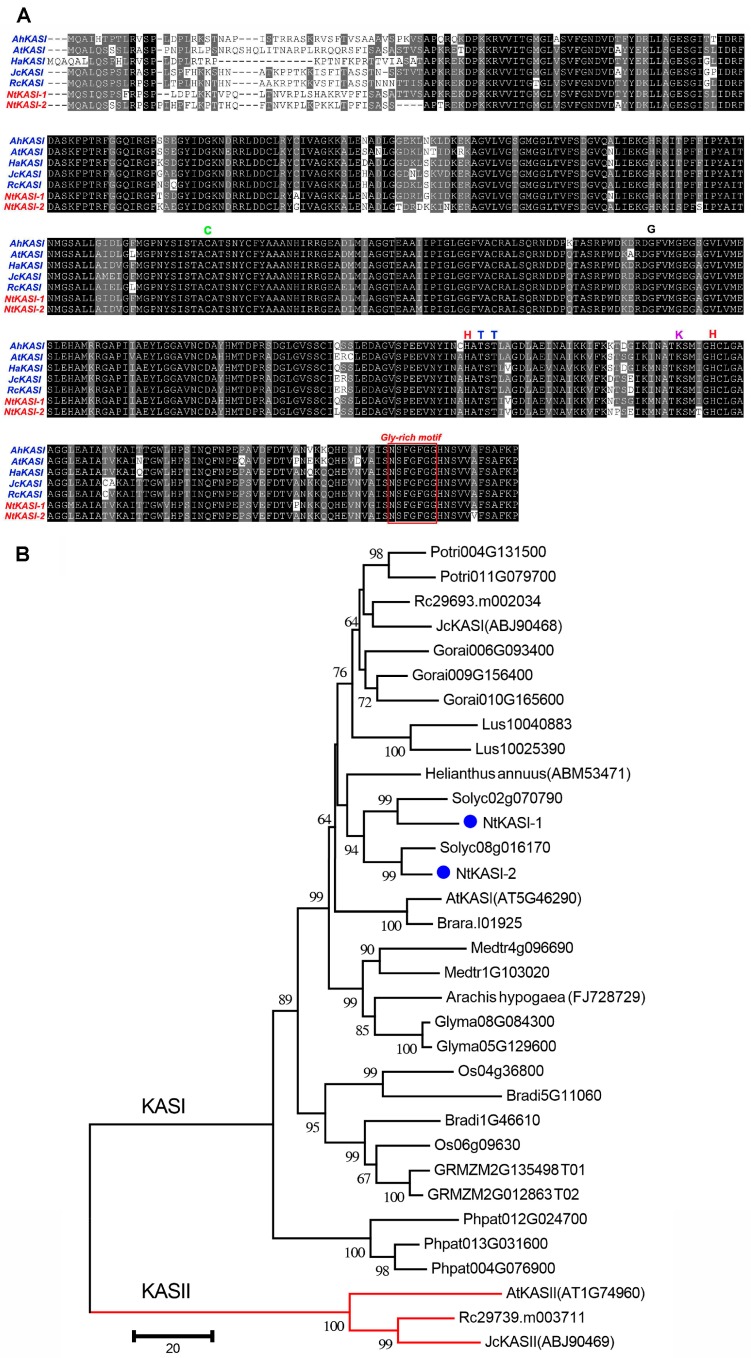
Multiple sequence alignment and phylogenetic analysis. (**A**) Comparison of amino acid sequences of KASI from different plants. The letters and red box represent the conserved residues and motif, respectively; (**B**) The phylogenetic tree of KAS proteins from diverse plants. The tree was generated with neighbor-joining methods and plant KASII proteins were used as the root of the KASI proteins phylogenetic tree. The blue dots represent the KASIs from tobacco. Potri, *Populus trichocarpa*; Rc, *Ricinus communis*; Jc, *Jatropha curcas*; Gorai, *Gossypium raimondii*; Lus, *Linum usitatissimum*; Solyc, *Solanum lycopersicum*; Nt, *Nicotiana tabacum*; At, *Arabidopsis thaliana*; Brara, *Brassica rapa*; Medtr, *Medicago truncatula*; Glyma, *Glycine max*; Os, *Oryza sativa*; Bradi, *Brachypodium sylvaticum*; GRMZM, *Zea may*; Phpat, *Physcomitrella patents.*

Multiple sequence alignments of amino acid sequences of KASI proteins from different plants revealed that KASIs were highly conserved in plants (Figure 1A). Some key functional sites were identified such as a substrate-binding cysteine (C) residue, two histidines (H) required for the decarboxylation, an essential lysine of uncertain function, one glycine (G) residue that allows entrance into the substrate-binding tunnel, and two threonine (T) residues that form hydrogen bond with the ACP phosphopantetheine moiety. In the C-terminal region, a conserved Gly-rich motif was also found that may act as forming oxide anion free radical [32]. The phylogenetic tree clearly demonstrated that NtKASI-1 and NtKASI-2 were clustered with two tomato KASIs (*Solanum lycopersicum*, which belongs to the same family as tobacco), respectively (Figure 1B), implying that *NtKASI-1* and *NtKASI-2* might have an independent evolution before the species differentiation between tobacco and tomato. Together, we isolated two *KASI* genes in tobacco with high sequences similarity, and whether these two genes have functional redundancy needs to be investigated.

### 2.2. Expression Patterns of NtKASI-1 and NtKASI-2

**Figure 2:**
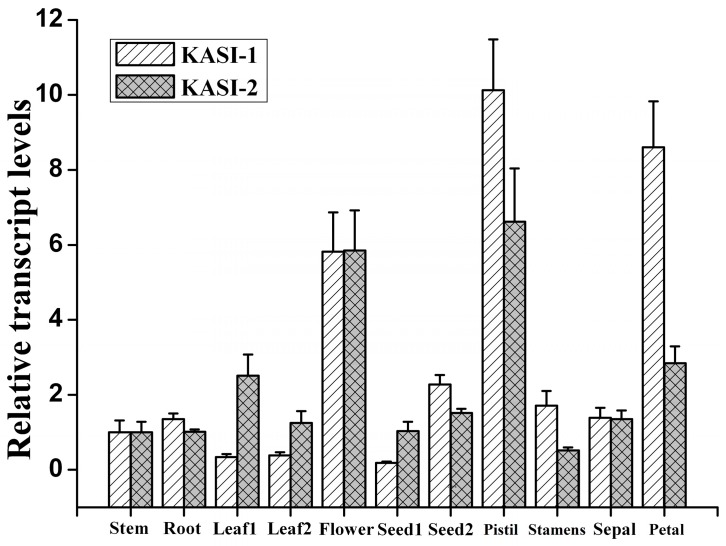
Relative transcript levels of tobacco *KASI* genes in different tissues by qRT-PCR. The relative expression was detected in root, flower, the 5th leaf at 60 DAG (leaf1), the 18th leaf at middle stage (leaf2), the seed at 6 DAF (seed1), and the seed at 12 DAF (seed2). Error bars show the standard error with five biological replicates. The expression level of stem was normalized to 1.

To gain insight into the possible roles of NtKASI-1 and NtKASI-2 in tobacco, we assayed their expression profiles in different tissues using a qRT-PCR technique. The results showed that both *NtKASI-1* and *NtKASI-2* were constitutively expressed in all tissues tested (Figure 2). It seemed that these two genes had nearly equal expression levels in stem, root, sepal and seed with low transcription abundance. Besides, *NtKASI-1* exhibited higher expression level than *NtKASI-2* in pistil, stamen and petal, whereas *NtKASI-2* was highly expressed in leaf relative to *NtKASI-1*. In sum, we found that *KASI* genes, particular for *NtKASI-1* gene, were highly transcribed in floral organ compared with vegetative tissues, implying their important function in reproductive stage.

### 2.3. Phenotypes of Over-Expression of NtKASI Genes in Tobacco

Generally, over-expression of a gene is considered as a genetic tool to dissect the gene function. Here*,* we constructed two binary plant transformation vectors harboring *NtKASI-1* and *NtKASI-2* gene, respectively, with a cauliflower mosaic virus (CaMV) 35S promoter, and transformed them into wild type (WT) tobacco, respectively. The leaves from T_0_ transformed plants were screened for successfully over-expressed lines via a hygromycin gene-specific primer PCR (Table S1). The seeds from transgenic T_0_ plants were collected and germinated in a medium with hygromycin selection. The confirmed over-expression lines in T_1_ generation for these two genes were designed as KASI-1OE lines (for *NtKASI-1* over-expressed) and KASI-2OE lines (for *NtKASI-2* over-expressed), respectively. Subsequently, the expression levels of *NtKASI* genes were examined and the phenotypic changes were investigated for each over-expressed plant.

**Figure 3:**
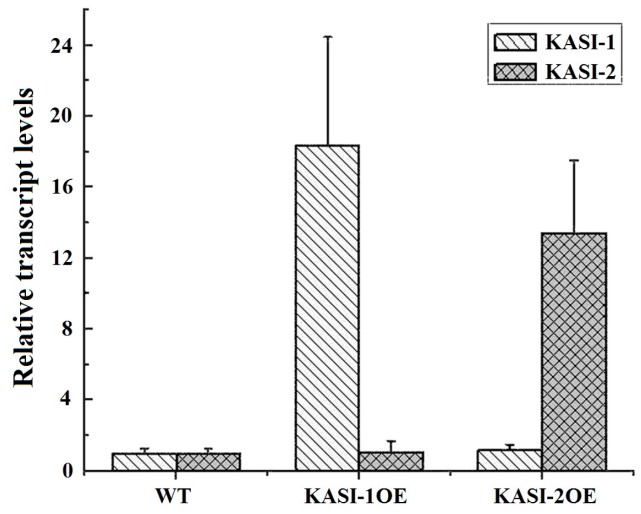
Relative transcript levels of *KASI* genes in all over-expressed lines and wild type (WT) tobacco. The expression of *NtKASI-1* and *NtKASI-2* gene in the 9th and 10th leaves in medium stage of wild type, KASI-1OE lines and KASI-2OE lines via qRT-PCR. The error bars represent the standard error with four independent lines.

**Figure 4:**
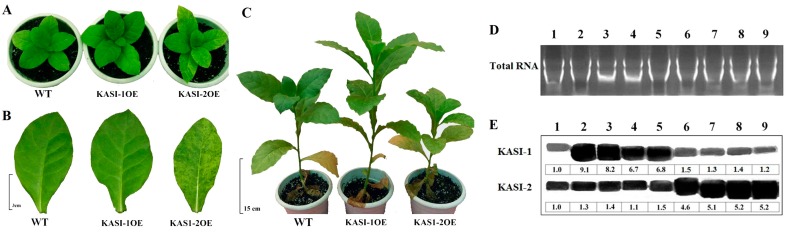
Phenotypic observation of *NtKASI* over-expression lines compared to WT plants: (**A**) phenotype at early-vegetative growth stage in WT, KASI-1OE and KASI-2OE line; (**B**) the leaf phenotype at early-vegetative growth stage (bar = 3 cm); (**C**) comparison of the growth at mid-vegetative growth stage (about four-month-old plants, bar = 15 cm); (**D**) total RNA were loaded on 1.2% agarose gels and stained with EtBr in buffer; and (**E**) Northern blot analysis for the expression of *NtKASI-1* and *NtKASI-2* gene in WT (Lane 1), KASI-1OE lines (Lane 2–5) and KASI-2OE lines (Lane 6–9).

As shown in Figure 3, the transcript levels of *KASI-1* in KASI-1OE transgenic lines were at least nine-fold (9–25-fold) higher than that of the WT plants, and *KASI-2* in KASI-2OE lines were at least eight-fold (8–20-fold) higher than that of the wild-type. Moreover, the expression levels of *NtKASI-1* were not affected in KASI-2OE lines and vice versa. Morphologically, all KASI-1OE lines grew much better, for example, there was a higher plant height than WT plants (Figure 4A–C). Interestingly, we found the opposite phenotype in KASI-2OE lines, such as variegated leaves and slightly dwarf (Figure 4A–C). We speculated that *NtKASI-1* or *NtKASI-2* transcript levels may be down-regulated in KASI-2OE lines via an RNAi way due to high sequence similarity between two *KASI* genes. To test this, the expression level of these two genes was subjected to Northern blot in all over-expressed lines and WT. We found that all the KASI-2OE plants displayed significantly high expression levels of the *NtKASI-2* gene compared to the WT plants, but the transcript level of *KASI-1* in leaf was not changed, consistent with the qRT-PCR result (see Figure 4D,E). These results showed that *NtKASI-1* gene can boost tobacco growth. However, for the KASI-2OE lines, the mechanism underlying the phenotypic changes remains unknown.

### 2.4. Silencing of NtKASI Genes in Tobacco

The gene silence or mutant is a classical genetic approach for exploring gene functions that cause a phenotype of interest. In the current study, we performed the RNA interference (RNAi) to obtain gene-silenced plants. As previously described, these two *NtKASI* genes had high similarities, but in the N-terminal region the sequences were more variable. Therefore, partial sequences from the 5′ of *NtKASI-1* (155 bp) and *NtKASI-2* (162 bp) ORF, where *NtKASI-1* and *NtKASI-2* have the greatest sequence difference, were cloned into the vector in an inverted repeat orientation to create RNAi constructs, namely pCXSN-KASI-1 RNAi (kasI-1 RNAi) and pCXSN-KASI-2 RNAi (kasI-2 RNAi), respectively. To obtain double silencing lines of *NtKASIs* (kasI-1/2 RNAi) in tobacco, we generated the construct using the identical sequence between *NtKASI-1* and *NtKASI-2* gene.

**Figure 5:**
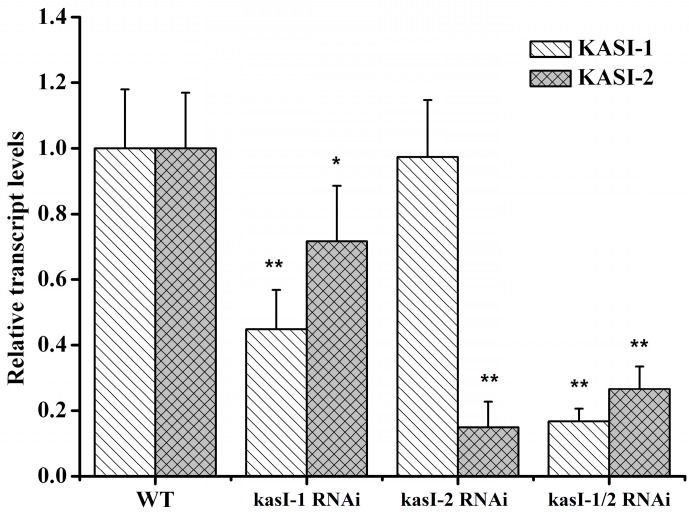
Relative transcript levels of *KASI* genes in the silence lines and WT tobacco. The expression of *NtKASI-1* and *NtKASI-2* gene in the 9th and 10th leaves in medium stage of wild type, kasI-1 RNAi, kasI-2 RNAi and kasI-1/2 RNAi lines using qRT-PCR. The error bars represent the standard error with four independent lines. The asterisk above the bars indicates the significant differences between the silence plants and WT (* *p* < 0.05, ** *p* < 0.01).

In the T_0_ generation, the silencing plants exhibited differentially phenotypic changes. For instance, 13 out of 20 independently transformed kasI-1 RNAi lines have kindly variegated leaves and semi-dwarf, whereas all kasI-2 RNAi plants of T_0_ generation were morphologically and developmentally similar to wild type. Furthermore, most of the double silence (kasI-1/2 RNAi) plants showed more obvious phenotypic changes and maldevelopment, such as severe variegated leaves, semi-dwarf and decreased seed production, and even death. Next, we collected the seeds from the *NtKASIs* silence plants and T_1_ generation seedlings were obtained for subsequent analysis. Similarly, the expression levels of *NtKASI-1 and NtKASI-2* were examined. The results showed that the expression of *NtKASI-1* was significantly decreased (approximately 70%) in kasI-1 RNAi lines, but there was a little decrease for the *NtKASI-2* gene (approximately 20%). In Si-2 lines, the expression of *NtKASI-2* exhibited a marked reduction (approximately 80%), but the *NtKASI-1* expression was not changed. In the double silencing lines, *NtKASI-1* (decreased approximately 85%) and *NtKASI-2* (decreased approximately 80%) were significantly co-silenced (Figure 5).

**Figure 6:**
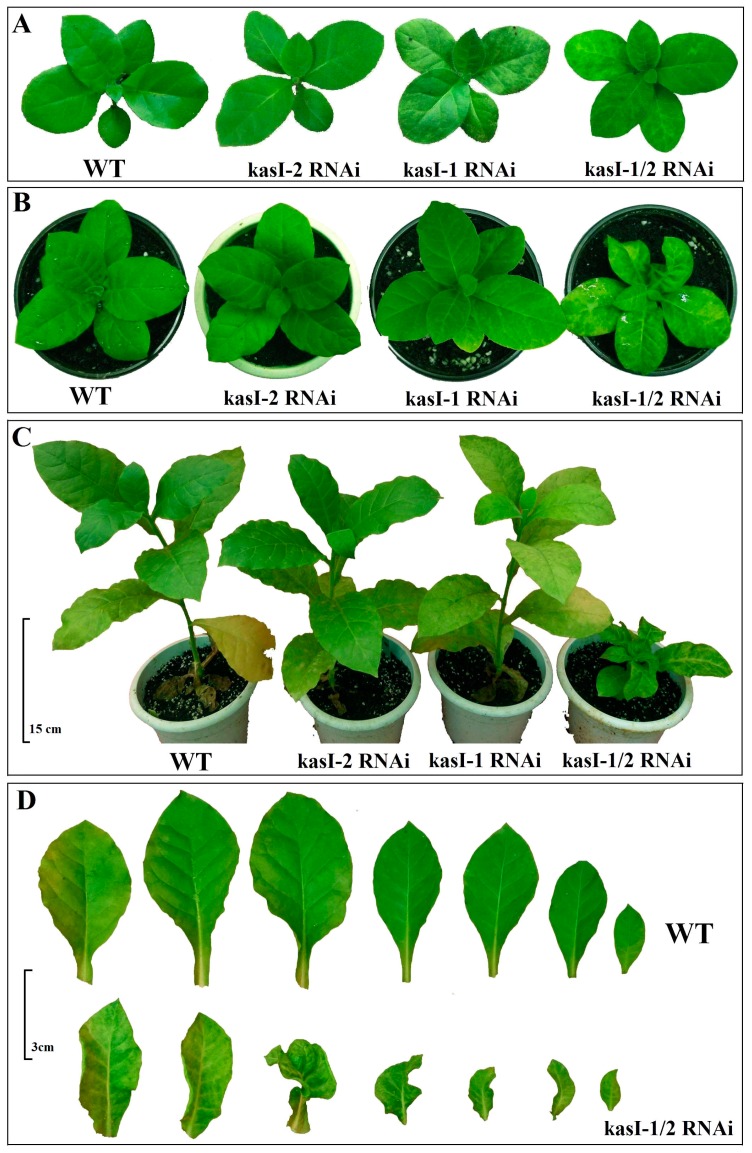
Growth and morphology of Wild-Type (WT) tobacco and kasI-1 RNAi, kasI-2 RNAi, kasI-1/2 RNAi plants: (**A**) phenotypic observation of two-month-old plants; (**B**) phenotypic observation of three-month-old plants; (**C**) phenotypic observation of four-month-old plants (bar = 15 cm); and (**D**) comparison of leaf morphology between WT plant and kasI-1/2 RNAi plant (three-months-old plant, bar = 3 cm).

Similar to phenotypes observed in the T_0_ plants, both kasI-1 RNAi and kasI-1/2 RNAi plants of T_1_ generation showed mildly variegated leaves in early stage (Figure 6A). During the leaf development, kasI-1/2 RNAi lines exhibited more serious growth defects such as loss of apical dominance, highly stunted stems and curled leaves, while kasI-1 RNAi showed mildly variegated leaves and stunted stem relative to WT plants (Figure 6B–D). However, the growth and development of kasI-2 RNAi plants was not affected compared to WT plants.

### 2.5. NtKASI Genes Affected the Chloroplast Development

**Figure 7:**
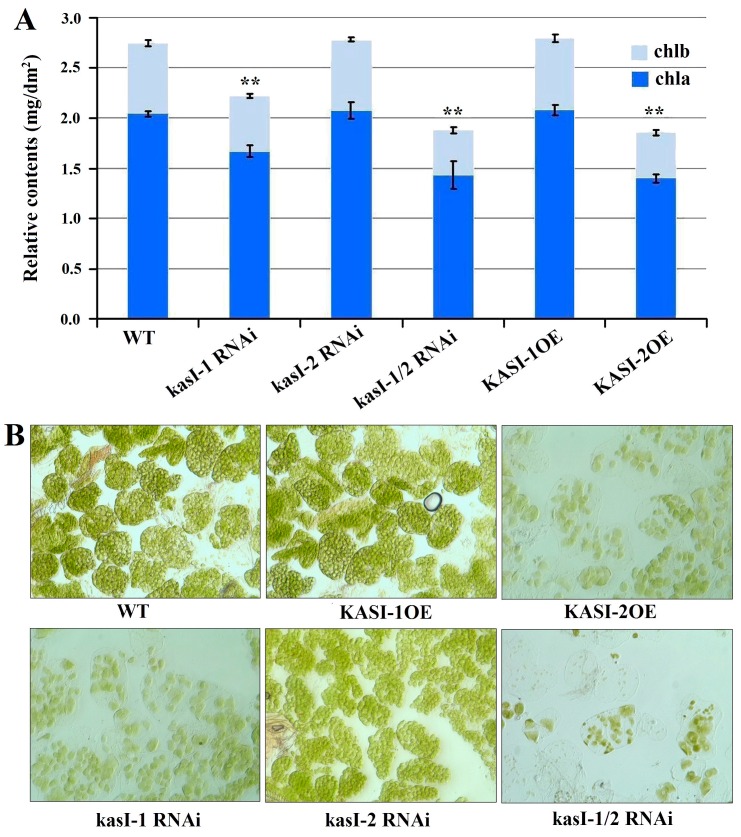
Measurement of chlorophyll content: (**A**) content of chlorophyll *a* and *b* (mg/dm^2^ fresh weight) measured in 8th, 9th and 10th leaves at four month after germination. The asterisks above the bar represent significant differences in content of chlorophyll *a* and *b* between transgenic lines and WT (** *p* < 0.01). Error bars represent the standard errors with four biological replications; (**B**) Morphological observation of chloroplast in leaves of WT and all transgenic lines.

It seems that the phenotype changes in all transgenic leaves were related to the chloroplast development. Thus, we investigated the chlorophyll (*a* and *b*) content in the leaves of the transgenic tobacco. We found that the content of chlorophyll *a* and *b* in transgenic lines of kasI-1 RNAi, kasI-1/2 RNAi and KASI-2OE lines was significantly decreased compared to the WT plants (Figure 7A), consistent with the variegated leaf phenotype, whereas the KASI-1OE and kasI-2 RNAi lines with normal development of leaf had similar levels of chlorophyll content relative to WT (Figure 7A). Further observation of chloroplast development in mesophyll cells using microscopy showed the presence of much fewer chloroplasts in the chlorotic sector of KASI-2OE, kasI-1 RNAi and kasI-1/2 RNAi leaves compared with many chloroplasts in wild type (Figure 7B). Consistent with the normal leaf phenotype, KASI-1OE and kasI-2 RNAi lines have no obvious defect in chloroplast development. These results showed that *NtKASI* genes may be involved into the chloroplast development.

### 2.6. NtKASI Genes Change Fatty Acids Composition in Tobacco Leaf

To determine whether tobacco NtKASIs affect the FAs biosynthesis, the total FA content and composition in leaves from all transgenic lines were examined. In over-expressed lines, we found that KASI-1OE plants did not result in the obvious increase of the total FA content compared to WT. As shown in Table 1, the content of FAs extracted from WT and KASI-1OE leaves was approximately 0.96 mg·g^−1^ and 1.03 mg·g^−1^ of fresh weight, respectively. However, the FA content in the KASI-2OE plants was remarkably reduced to 0.76 mg·g^−1^ of fresh weight, similar to transgenic silence plants (0.76 mg·g^−1^ in kasI-1/2 RNAi), resulting in a decrease of 21% relative to WT plants. Besides, the kasI-1 RNAi and kasI-2 RNAi lines also showed a significant decrease of total FA content in leaves.

We further performed the gas chromatographic (GC) analysis for detecting the FA profiles in all transgenic tobacco and WT leaves. In this study, we classified the FA species into three families according to the length of the carbon chain, namely medium-chain FAs (10C–14C), long-chain FAs (16C–18C) and very-long-chain FAs (>18C). Interestingly, although the FAs content was not changed notably in KASI-1OE lines, the FA composition was altered by the decrease of medium-chain and very long-chain FAs proportions and an increase of long-chain FAs proportions compared to WT plants. A similar change in FA profile was also observed in KASI-2OE lines. By the contrast, kasI-1/2 RNAi lines showed a significant increase in medium-chain FAs from 11.09% to 16.96%, and the reduction of long-chain FA proportions from 85.6% to 78.34%. Accompanied by these changes, all transgenic lines showed a dramatic change in very-long-chain FAs. The unsaturated FAs to saturated FAs ratio (US/S) showed a most significant reduction in kasI-1/2 RNAi. Taken together, our findings showed that the silencing of *NtKASI* decreases the total FA content while increases the medium-chain FAs ratio.

### 2.7. NtKASI Genes Affect the Seed Weight and Lipid Content

**Figure 8:**
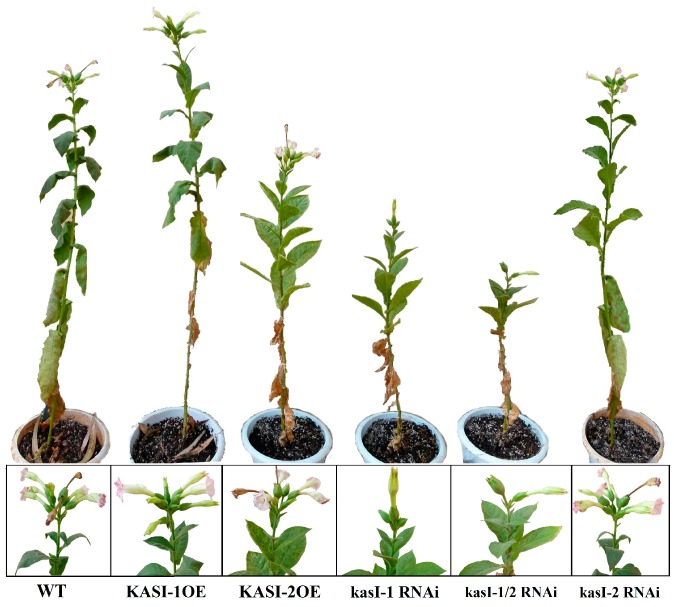
Phenotype of reproductive growth.

In T_1_ plants, the transgenic lines showed phenotypic differences during the reproductive growth stage. In particular, due to the serious maldevelopment of kasI-1/2 RNAi lines during vegetative growth stage only about 20% of the transgenic plants produced flowers in the end. The kasI-1/2 RNAi plants exhibited significantly low fruiting rate with a mean flower number of 2 compared to WT lines with a mean flower number of 9 (Figure 8). The KASI-2OE (mean flower number of 6) and kasI-1 RNAi lines (mean flower number of 3) also exhibited relatively low fruiting rates, whereas the KASI-1OE and Si-2 lines showed a non-significant difference in fruiting rate compared to WT plants (Figure 8).

**Figure 9:**
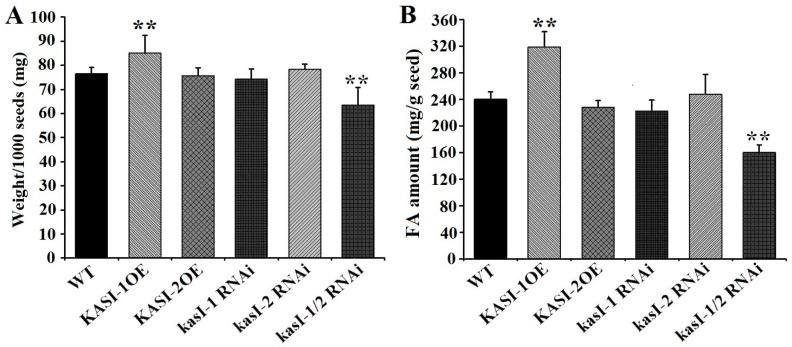
Seed weight and lipid content in tobacco: (**A**) thousand-seed weights in WT and all transgenic lines; and (**B**) the lipid contents in WT and all transgenic lines. Error bars represent the standard errors with four biological replications. The asterisk above the bars indicates the significant differences between transgenic plants and WT (** *p* < 0.01).

We then examined whether seed weight, oil content and FA composition were changed in all transgenic lines. As shown in Figure 9A, measurements of seed weight revealed that the *NtKASI-1* over-expressing lines had significantly higher thousand-seed weights than WT plants. In contrast, kasI-1/2 RNAi double silence lines exhibited markedly lower thousand-seed weights than WT plants. Other three transgenic lines (kasI-1 RNAi, kasI-2 RNAi and KASI-2OE) had no obvious difference in seed weights (Figure 9A). The lipid content in seeds of wild plants and various transgenic lines was also measured. Similar to seed weights, the KASI-1OE lines seeds had high levels of lipid content, whereas the kasI-1/2 RNAi seeds had low levels of lipid content relative to WT lines. Contents of total FA in the kasI-1 RNAi, kasI-2 RNAi and KASI-2OE lines seeds had no obvious changes compared to WT seeds (Figure 9B). As to FA composition, levels of all FA species from 16C–22C showed a non-significant change in all transgenic lines (Table S2). These results indicated that *NtKASI* genes significantly influenced the tobacco seed weight as well as lipid content, particularly for *NtKASI-1* gene.

## 3. Discussion

In plants, FAs are used for the synthesis of plastid and other cellular membranes in all cells and are also converted for producing various plant hormones, participating in regulating plant growth and development, cell signaling [33,34,35] and stress responses [33,36]. As mentioned above, Wu and Xue [10] firstly characterized the functions of KASI in regulating FAs biosynthesis and found that it affects multiple developmental processes such as altered chloroplast division and suppressed embryo development in *Arabidopsis*. Similarly, the mutant of rice *KASI* (*OsKASI*) also resulted in a remarkable change in fatty acid (FA) composition and contents and reduced fertility. In addition, OsKASI also is involved in regulating the root development in rice [28]. Generally speaking, the functions of KASI have major roles in controlling FAs biosynthesis and regulating growth and development in plants.

In the current study, we isolated two NtKASI orthologs from *Nicotiana tabacum* that were highly conserved among various plants. Similar to their functions in *Arabidopsis*, the knock-down *NtKASI-1* (kasI-1 RNAi lines) plants exhibited the variegated leaves and semi-dwarf phenotype. Moreover, the knock-down of both *NtKASI-1* and *NtKASI-2* (kasI-1/2 RNAi line) caused more seriously variegated leaves and a significant decrease in chlorophyll content, resulting in the vegetative stage maldevelopment compared to the kasI-1 RNAi lines and WT plants. These results imply that there is a partial functional redundancy between *NtKASI-1* and *NtKASI-2*, and particularly *NtKASI-1* has a stronger role in regulating growth and development in tobacco. In *Arabidopsis*, the mutant of *KASI* suppressed the expression of *FtsZ* and *Min* system genes, resulting in abnormal development of chloroplast division [10]. This implied that the similar mechanism of KASI may be present in tobacco leaf and needs to be investigated. In addition to abnormal vegetative growth, the *NtKASI* kasI-1/2 RNAi lines caused the reduction of plant height, seed weights and fertility during the reproductive stage similar to the mutation in OsKASI [28]. The higher expression of *NtKASI* genes in floral organs, especially pisitil and petal, strongly implied that *NtKASI* involved in regulating the flower development. Thus, it seems to be not difficult in an understanding of the flower development defect in silenced *NtKASI* lines. Besides, many evidences have shown that lipid genes involved in FA biosynthesis affected the vegetative and reproductive growth of plants. For instance, the knock-out of genes *FATB* (acyl-ACP thioesterases B), a major regulator for controlling saturated FAs fluxes, suppressed the rosettes size and delayed the bolting in *Arabidopsis* [1]. Interestingly, over-expressed *NtKASI-2* plants exhibited a similar phenotype with the silenced *NtKASI-1*. Initially, we proposed that the over-expressed *NtKASI-2* might result in the reduction of *NtKASI-1* or *NtKASI-2* expression via post-transcriptional gene silencing. However, this assumption should be ruled out because there was no obvious change of both *NtKASI-1* and *NtKASI-2* gene expression levels, which was confirmed by qRT-PCR and Northern blot tests. Thus, the potential mechanism of an *NtKASI-2* gene in the control of the plant growth and development remains unknown in tobacco.

The deficient *NtKASI* genes result in dramatic changes of FA content and profiles in tobacco leaves, consistent with the observation in *Arabidopsis* [10]. In leaf, the medium-chain FAs (10C-14C) in deficient *NtKASI-1* and kasI-1/2 RNAi lines have a significant increase, suggesting that the elongation of FA chains be affected by deficient *NtKASI*s. In addition, the reduction in the content of unsaturated fatty acids in the kasI-1/2 RNAi line (see Table 1) means that *NtKASI-1* and *NtKASI-2* might participat in the processes of FA desaturation. The reduction of unsaturated/saturated FAs ratio (US/S) may be related to the variegated leaf phenotypes in kasI-1 RNAi and kasI-1/2 RNAi lines. Usually, the de novo FA synthesis provides FA sources for the normal membrane formation, which is necessary for maintaining the normal cell growth and development. The changes of unsaturated/saturated FAs ratios in leaves may be the potential cause of developmental abnormalities observed in the variegated and curled leaves and reduction in chloroplast division. Higher levels of saturated FAs may cause the formation of semi-crystalline gels in cell membranes, which impair the cell permeability thereby causing the leaf maldevelopment [37]. Besides, there are evidence showing that very long chain fatty acids (>C18) usually provide FA sources for maintaining plant growth, cell expansion and ethylene biosynthesis and signaling [2,38]. In addition, the leaf variegated in the kasI-1 RNAi and kasI-1/2 RNAi lines might be related to the increase proportion of very long chain fatty acids in leaf FA species. The over-expressed *NtKASI-1* plants (in KASI-1OE line) slightly but not significantly increased the FA content in leaves, implying that some other factors are also required for enhancing FAs accumulation. As mentioned above, FAs synthesis is a complex process involving diverse enzymes and regulators, in particular, the rate limiting enzyme acetyl-CoA carboxylase responsible for the synthesis of malonyl-CoA [39,40].

Seed oils have been regarded as the potential feedstock for chemical industries and biodiesel production due to the raised concern of developing renewable and environment-friendly alternatives for crude oil [4,5]. As the initial carbon chain condensation enzyme of the de novo biosynthesis of FAs, KAS enzymes are indispensable for FA chain elongation. Usually, the over-expressed *KAS* genes positively influence the subsequent TAG assembly and oil content. Here, we also evaluated the specific roles of NtKASI-1 and NtKASI-2 in TAG biosynthesis in tobacco seeds. The results showed that the over-expressed *NtKASI-1* significantly enhanced oil content and seed weight, but the silenced *NtKAkasI-1/2 RNAi* genes significantly decreased the oil content and seed weights, clearly suggesting that *NtKASI-1* might play a critical role in regulating TAG accumulation in tobacco seeds. In addition, we noted that the silenced *NtKASI-1* (kasI-1 RNAi lines) did not exhibit a significant reduction in the oil content and seed weight. Probably, this is related to the functional compensation of *NtKASI-2* in the silenced *NtKASI-1* plants. Besides, the over-expressed *NtKASI-1* (KASI-1OE line) obviously promoted plant vegetative growth, which might provide more photosynthate feedstock in leaf for TAG biosynthesis in seeds. In addition, the FA profiles of seed oils among the transgenic lines and WT control did not display a significant difference, unlike FA composition’s dramatic change in leaves. Although the potential reasons remain uncertain, our current results are largely consistent with previous reports in which modifying the expression levels of genes involved in de novo fatty acid biosynthesis did not exert a significant change in fatty acids composition in seed oils [41,42,43].

## 4. Materials and Methods

### 4.1. Isolation and Sequence Analysis of Tobacco KASI Genes

In order to isolate the putative *KASI* genes in tobacco, we performed a BLASTN search using the CDS sequence of *Arabidopsis* *KASI* (At5g46290) in all tobacco EST (Expression Sequence Tag) database (NCBI; http://www.ncbi.nlm.nih.gov/dbest/). The fragments with high similarity score were collected and assembled. The full-length fragments of tobacco *KASI* genes were further confirmed by RT-PCR sequencing. Briefly, tobacco (*Nicotiana tabacum*) young leaves were harvested for RNA extraction using TaKaRa RNA extraction kit (TaKaRa, Dalian, China) with the manufacturer’s protocol. The first strand cDNA was synthesized from 1 μg of total RNA using a PrimeScript^TM^ RT-PCR Kit (TaKaRa, Dalian, China). The full lengths of ORF (open reading frame) were amplified using high fidelity PCR TransStart FastPfu DNA Polymerase (TransGen, Beijing, China) with gene-specific primers (see Table S1). The PCR products were cloned into pEASY-Blunt Cloning Vector (TransGen, Beijing, China) and sequenced (shenzhen-BGI, Shenzhen, China). Sequence similarity was analyzed by the BLAST search in GenBank and a multiple sequences alignment in CLASTAL W program [44]. Phylogenetic analysis was conducted using the Neighbor-Joining criteria in MEGA (version 5.0) [45]. Branch support of the phylogenetic tree was estimated on the basis of 10,000 bootstrap replicates of the data.

### 4.2. Vector Construction and Transformation

For two *KASI* genes confirmed, we constructed two plant over-expression PCXSN vectors [46] using ORF sequence, respectively. Meanwhile, three RNAi transcriptional silencing vectors were designed using partial sequences of each *KASI* in an inverted-repeat fashion to create each gene suppressed (RNAi-KASI-1, RNAi-KASI-2) and both genes suppressed (RNAi-KASI-1/2) transformants. The primers used are listed in Supplementary Table S1. The authenticities of all recombinant vectors were verified by sequencing. Then, all vectors were transferred into tobacco using the Agrobacterium-mediated tobacco (*Nicotiana tabacum*) leaf disc transformation method [47] with a hygromycin (20 mg/L) selection. Transgenic individuals obtained were grown in a phytotron with 25 °C under a switch of 16-h-light/8-h-dark. The transgenic individual was confirmed by PCR with hygromycin-specific primers (see Table S1), and was moved to the greenhouse for further growth.

### 4.3. Expression Analyses of KASI Genes in Wild-Type and Transgenic Tobacco

To inspect the expression profiles of *NtKASI-1* and *NtKASI-2* in wild-type (WT) tobacco, the different tissues including root, leaf, stem, pistil, stamen, sepal, petal tissues and developing seeds were collected. Total mRNAs were extracted from these tissues and were reversely transcripted using PrimeScript^TM^ RT reagent Kit with gDNA Eraser (TaKaRa, Dalian, China). Real-time PCR analysis was performed according to the SYBR Premix Ex Taq^TM^ (Tli RNaseH Plus, TaKaRa, Dalian, China) manufacturer’s manual. For transgenic tobacco plants (T_0_), the qRT-PCR was also performed to test the expression levels of *KASI* genes using the mRNA from a young leaf. Quantitative RT-PCR was performed using the CFX96 machine (Bio-Rad, Hercules, CA, USA) following the manufacturer’s instructions. The amplification program was 95 °C for 10 s and 56 °C for 20 s. The relative quantification of each sample was determined by normalization to the amount of *NtActin* cDNA detected in the same sample. Primers used for quantitative RT-PCR were listed in Supplementary Table S1.

Further, Northern blot analysis was performed on a 1.2% (*w*/*v*) agarose gel to confirm the gene expression levels of *KASI* genes in transgenic plants. Ethidium bromide staining was used to ensure the equal loading. The mRNA was then transferred to Hybond N^+^ nylon membranes (Amersham Pharmacia Biotech, Piscataway, NJ, USA) and fixed by drying at 80 °C for 1 h. mRNA gel blots were hybridized with DIG-dUTP-labeled probe prepared from the cDNA of the tobacco *KASI* genes using Roche PCR DIG Probe Synthesis Kit (Roche Cat. No. 1636090) (primer sequences were listed in Supplementary Table S1). Before addition to the filters in the hybridization solution, probes were denatured by dipping in boiling water for 5 min and then in ice. Pre-hybridization was done in 6× SSC (1× SSC is 150 mM NaCl and 15 mM sodium citrate), 5× Denhardt’s solution, 0.5% (*w*/*v*) SDS and 100 μg·mL^−1^ denatured salmon sperm DNA at 50 °C for 30 min, while the hybridization was done in 6× SSC, 0.5% (*w*/*v*) SDS and 100 μg·mL^−1^ denatured salmon sperm DNA at 50 °C for overnight. Afterward, the blot was subsequently washed in 2× SSC, 0.5% (*w*/*v*) SDS twice and in 0.5× SSC, 0.5% (*w*/*v*) SDS once, then exposed to X-ray film. Hybridization was visualized by autoradiography after exposure. Hybridization was performed using the DIG Northern starter kit (Roche Cat. No. 2039672). The gene-specific primers for detecting transcripts of *NtKASI-1* and *NtKASI-2* were listed in Supplementary Table S1.

### 4.4. Morphological Observation of Chloroplast and Measurement of Chlorophylls

Chloroplast morphology of mesophyll cells was observed by OlympusBX51 microscope. Seventh and eighth rosette leaves at four months were collected. For all plants, The leaves of the same size were separated by a hole puncher and fixed in 3.5% glutaraldehyde in the dark for 60 min, and then were placed in 0.1 M Na_2_EDTA, pH 9.0, for overnight to allow the fixative to soften and then incubated at 60 °C with shaking for 2 to 3 h [48]. After that, the tissues were mounted in water, and cells were released by tapping on the cover slip. The chloroplasts were observed directly on the slide. Images were captured with ZEISS Discovery.V12 digital camera.

Amounts of chlorophyll *a* and *b* were quantified using a simple method. Leaf filaments with known areas are soaked in 80% (*v*/*v*) acetone until the color of filaments changed from green to white, and then the supernatant is taken for light absorption measurement [49].

### 4.5. Seed Weight Determination and Lipid Analysis

To examine seed weight, four replicates of 100 tobacco seeds randomly selected from WT and transgenic lines were weighted. These seeds were dried in open tubes in desiccators for 3 days before weighing and counting. Total lipids were extracted from leaves and mature seeds of different transgenic lines and WT plants according to the method of Bligh and Dyer (1959) [50], and then fatty acid methyl esters (FAMEs) were prepared according to the description by Maisonneuve et al. (2010) [51]. In brief, fatty acids of total lipid were transmethylated with 2 mL of methanol containing 2.5% H_2_SO_4_ (*v*/*v*) and then heating at 85 °C for 90 min. In addition, we used a heptadecanoic acid as an internal standard at a final concentration of 50 ng·μL^−1^ for quantification. After cooling, 500 μL of hexane and 2.5 mL of 500 mM Na_2_SO_4_ were added. FAMEs were extracted into the hexane phase by vigorous shaking followed by centrifugation at 1500× *g* for 5 min. FAMEs were quantified by gas chromatography (Agilent 5975 system with an HP-INNOWax column). Individual methyl esters were identified by comparison with standards (Sigma-Aldrich, Shanghai, China). FAMEs and total lipids were calculated by comparing with the heptadecanoic acid methyl ester standard.

## 5. Conclusions

In conclusions, we revealed that the *NtKASI* genes, particularly *NtKASI-1*, are crucial for regulating fatty acids synthesis in leaf and seeds, and play a key role in tobacco vegetative and reproductive growth. This work might establish a good basis for further studies on dissecting the functions of *KASI* genes in regulating oil accumulation in specific plant tissues, serving for modifying oil content and quality via genetic improvement and breeding in oil crops.

## Figures and Tables

**Table 1:** Analysis of fatty acid methyl esters (FAME) of the total lipid extract from leaves of wild type (WT) and transgenic tobacco.

FA Species	WT	KASI-1OE	KASI-2OE	kasI-1 RNAi	kasI-2 RNAi	kasI-1/2 RNAi
10C	6.07 ± 0.48	4.66 ± 0.66	4.33 ± 0.65	6.56 ± 0.40	5.29 ± 0.28	4.39 ± 0.41
12C	3.31 ± 0.35	2.77 ± 0.35	2.44 ± 0.51	3.13 ± 0.75	3.34 ± 0.25	11.06 ± 0.61
14C	1.71 ± 0.56	1.67 ± 0.54	2.43 ± 0.60	1.81 ± 0.67	2.34 ± 0.11	1.51 ± 0.20
Total	11.09	9.10	9.20	11.50	10.97	16.96 **
16C	11.07 ± 1.30	13.66 ± 1.83	11.66 ± 1.08	11.43 ± 0.18	11.32 ± 0.49	11.97 ± 2.38
16C1	2.80 ± 0.16	2.47 ± 0.70	2.08 ± 0.24	2.93 ± 0.13	2.56 ± 0.34	1.16 ± 0.31
16C2	8.88 ± 1.44	8.34 ± 0.12	8.72 ± 0.50	9.02 ± 0.14	8.66 ± 1.20	6.51 ± 0.23
16C3	2.14 ± 0.22	3.32 ± 0.30	2.90 ± 0.27	2.26 ± 0.16	2.05 ± 0.52	1.10 ± 0.10
18C	2.12 ± 0.22	3.76 ± 0.32	2.98 ± 0.28	2.63 ± 0.15	2.45 ± 0.14	3.07 ± 0.49
18C1	3.73 ± 0.13	4.73 ± 0.24	4.10 ± 0.20	4.19 ± 0.14	4.06 ± 0.19	4.51 ± 0.43
18C2	11.48 ± 1.26	10.49 ± 0.34	12.57 ± 0.25	12.76 ± 0.16	12.36 ± 0.90	14.04 ± 3.55
18C3	43.38 ± 1.34	43.50 ± 1.11	44.35 ± 1.31	38.63 ± 1.95	43.86 ± 1.29	35.98 ± 2.52
Total	85.60	90.27 *	89.36	83.85	87.32	78.34 **
20C	2.74 ± 0.51	1.45 ± 0.60 **	1.43 ± 0.22 **	5.55 ± 0.19 **	1.71 ± 0.61 *	4.7 ± 0.75 **
US/S	2.7	2.6	3.0	2.2	2.7	1.7
FA content	0.96 ± 0.13	1.03 ± 0.09	0.76 ± 0.05 **	0.83 ± 0.03 *	0.88 ± 0.07	0.76 ± 0.07 **

Numbers in each column refer to the relative molar ratios of the different FA with the total being 100%. Means and standard deviation of four independent samples are presented; US/S refers to the ratio of unsaturated/saturated FA. FA content was measured by mg/g fresh weight. The asterisk indicates significant difference in fatty acids composition between transgenic plants and WT (* *p* < 0.05, ** *p* < 0.01).

## Supplementary Materials

Supplementary materials can be found at www.mdpi.com/1422-0067/17/8/1287/s1.

## Abbreviations

FA	Fatty Acid
ACCase	Acetyl-CoA Carboxylase
CoA	Coenzyme A
ACP	Acyl Carrier Protein
KAS	3-Ketoacyl-Acyl-Carrier Protein Synthase
TAG	Triacylglycerol
GC	Gas Chromatographic
